# FABP7 in Hepatic Macrophages Promotes Fibroblast Activation and CD4^+^ T-Cell Migration by Regulating M2 Polarization During Liver Fibrosis

**DOI:** 10.1155/jimr/6987981

**Published:** 2025-02-19

**Authors:** Hirofumi Miyazaki, Tunyanat Wannakul, Shuhan Yang, Dandan Yang, Ayano Karasawa, Ai Shishido, Ruizhu Cao, Yui Yamamoto, Yoshiteru Kagawa, Shuhei Kobayashi, Masaki Ogata, Motoko Maekawa, Yuji Owada

**Affiliations:** ^1^Department of Organ Anatomy, Graduate School of Medicine, Tohoku University, Sendai, Miyagi, Japan; ^2^Department of Anatomy, Tohoku Medical and Pharmaceutical University, Sendai, Miyagi, Japan; ^3^Florey Institute of Neuroscience and Mental Health, University of Melbourne, Melbourne, Victoria, Australia; ^4^Department of Immunology, Kanazawa Medical University, Uchinada, Ishikawa, Japan; ^5^Fukushima Institute for Research, Education and Innovation, Namie, Fukushima, Japan

**Keywords:** FABP7, hepatic macrophages, liver fibrosis, macrophage polarization

## Abstract

Hepatic macrophages respond to various microenvironmental signals and play a central role in maintaining hepatic homeostasis, dysregulation of which leads to various liver diseases. Fatty acid-binding protein 7 (FABP7), an intracellular lipid chaperone for polyunsaturated fatty acids (PUFAs), is highly expressed in liver macrophages. However, the mechanisms by which FABP7 regulates hepatic macrophage activation remain unclear. Therefore, we aimed to elucidate the mechanisms underlying the effects of FABP7 on the functions of hepatic macrophages in metabolic dysfunction-associated steatohepatitis (MASH) and liver fibrosis models. In this study, we found that FABP7-deficient macrophages exhibited impaired M2 polarization, which reduced the fibrotic response of myofibroblasts and CD4^+^ T-cell infiltration into the liver tissues in a carbon tetrachloride (CCl_4_)-induced hepatic fibrosis model. In vitro, FABP7-deficient macrophages exhibited decreased levels of peroxisome proliferator-activated receptor (PPAR)-*γ* and its target genes, including C–C motif chemokine ligand (CCL)-17 and transforming growth factor-*β* (TGF-*β*), compared to the wild-type (WT) macrophages post-interleukin (IL)-4 stimulation. However, these effects were inhibited by a PPAR*γ* inhibitor. IL-4-stimulated WT macrophages also promoted CD4^+^ T-cell migration and hepatic fibroblast (TWNT-1 hepatic stellate cell [HSC]) activation, indicated by increased mRNA levels of actin alpha 2, smooth muscle (*ACTA2*), and collagen type I alpha 1 (*COL1A1*); however, these effects were inhibited in FABP7-deficient macrophages. Overall, FABP7 in hepatic macrophages modulated the crosstalk between hepatic fibroblasts and T cells by regulating M2 polarization. Therefore, regulation of hepatic macrophage function by FABP7 is a potential therapeutic target for liver fibrosis.

## 1. Introduction

Macrophages, innate immune cells present in almost all tissues of the body, play key roles in the maintenance of tissue homeostasis via cytokine production and phagocytosis. Additionally, they play crucial roles in various pathophysiological processes, such as inflammation, tumorigenesis, tissue repair, and metabolism [1–3]. Upon activation, they differentiate into different phenotypes in response to microenvironmental conditions or changes in a process, known as macrophage polarization. Polarized macrophages are generally classified as pro-inflammatory (classically activated, M1) or anti-inflammatory (alternatively activated, M2) macrophages. Lipopolysaccharides (LPS) and interferon-*γ* (IFN-*γ*) induce M1 macrophages to promote inflammation, eliminate pathogenic microorganisms, and exert antitumor effects, whereas interleukin (IL)-4 and IL-13 induce M2 macrophages to suppress inflammation, tissue remodeling, angiogenesis, immune regulation, and tumor progression [2]. However, considering the complex regulation by various stress signals, such as cytokines, growth factors, damage-associated molecular patterns, pathogen-associated molecular patterns, eicosanoids, leukotrienes, fatty acids, and cholesterol, the mechanisms underlying M1/M2 polarization in macrophages remain controversial.

Hepatic macrophages, such as Kupffer cells (KCs) and monocyte-derived macrophages, are involved in liver homeostasis by removing pathogenic substances derived from the intestinal tract and phagocytosing the damaged old or dead blood cells. Macrophages are also involved in the pathogenesis of various liver diseases. For instance, M1-polarized hepatic macrophages aggravate, whereas M2-polarized hepatic macrophages alleviate metabolic dysfunction-associated steatohepatitis (MASH), formerly known as nonalcoholic steatohepatitis (NASH) [4, 5]. Liver fibrosis, which is characterized by the deposition of excess extracellular matrix (ECM) produced by activated hepatic stellate cells (HSCs), is a risk factor for the progression of cirrhosis and hepatocellular carcinoma. Hepatic macrophages activate HSCs, promoting liver fibrosis progression, and drive HSC apoptosis and ECM degradation during recovery from liver fibrosis [6]. Therefore, understanding the mechanisms regulating hepatic macrophage function is important to develop effective therapies.

Fatty acid-binding proteins (FABPs) are intracellular chaperones of long-chain fatty acids that regulate lipid metabolism, signal transduction, and gene expression [7, 8]. Many studies have investigated the expression patterns and physiological and pathophysiological functions of FABPs in macrophages [8, 9]. FABP4 (known as adipocyte FABP, A-FABP, or aP2), expressed in foamed macrophages, is associated with atherosclerosis in apolipoprotein E-deficient mice [10, 11]. FABP4 is also expressed in M2-like tumor-associated macrophages and plays a critical role in pro-tumor activity by promoting the IL-6/signal transducer and activator of transcription (STAT)-3 signaling pathway in a murine breast cancer model [12]. FABP5 (epidermal FABP, E-FABP, or Mal-1) is ubiquitously expressed in macrophages and controls (CTs) alternative macrophage activation by modulating the metabolism of long-chain unsaturated fatty acids [11, 13, 14]. Therefore, FABPs act as functional markers defining macrophage function and are crucial for maintaining homeostasis in the body.

Previously, we demonstrated that liver macrophages exhibit high levels of FABP7 without FABP4 or FABP5 expression [15, 16]. We also demonstrated impaired phagocytic function of liver macrophages during acute liver injury and reduced levels of liver fibrosis induced by carbon tetrachloride (CCl_4_) in *Fabp7*-deficient mice compared to those in wild-type (WT) mice [17]. However, the precise mechanisms by which FABP7 regulates hepatic macrophage polarization in liver diseases, such as MASH and liver fibrosis, remain largely unknown. Therefore, in this study, we aimed to elucidate the mechanisms underlying the effects of FABP7 on the functions of hepatic macrophages in MASH and liver fibrosis models. We found that FABP7 deficiency in macrophages impaired M2 polarization, subsequently inhibiting the fibrotic response of myofibroblasts and infiltration of CD4^+^ T cells into the liver tissues.

## 2. Materials and Methods

### 2.1. Mice

Male WT and *Fabp7*-gene knockout (*Fabp7^−^*^/−^) [16] C57BL/6 mice were used in this study. All experimental procedures were reviewed and approved by the Ethics Committee for Animal Experimentation, adhered to the Guidelines for Animal Experimentation of the Tohoku University School of Medicine, and complied with the regulations stipulated by the Japanese Government.

### 2.2. Establishment of a Mouse Liver Fibrosis Model

Male WT and *Fabp7*^−/−^ [16] C57BL/6 mice at 8–12 weeks of age were induced via intraperitoneal injection of CCl_4_ at a dose of 1 μL/g body weight diluted 1:3 in olive oil. CCl_4_ was administered 16 times over 8 weeks (twice weekly) to establish a hepatic fibrosis model. In the CT group, phosphate-buffered saline (1 μL/g body weight diluted 1:3 in olive oil) was administered intraperitoneally using the same method described above. Four groups (WT-CCl_4_, *Fabp7^−^*^/−^ CCl_4_, WT-CT, and *Fabp7^−^*^/−^-CT) were analyzed. One investigator divided the mice into four groups, and another investigator administered CCl_4_ phosphate-buffered saline to the mice to establish hepatic fibrosis and CT models in a blinded manner.

### 2.3. Hepatic Macrophage Isolation

Mouse livers were digested using the Liver Dissociation Kit (130-105-807; Miltenyi Biotec) with gentle-MACS Dissociators, following the manufacturer's instructions. Liver cells were stained with fluorescently labeled antibodies and analyzed using BD FACSAria II (BD Biosciences). Hepatic macrophages were isolated (Figure S1) to extract mRNA for quantitative real-time polymerase chain reaction (PCR) analysis. All antibodies used here are listed in Table S1.

### 2.4. Bone Marrow-Derived Macrophage (BMDM) Culture and Stimulation

BMDMs were harvested as previously described [18], with some modifications. Bone marrow cells were collected from the femurs and tibias of 8–10-week-old WT and *Fabp7^−^*^/−^ mice. Subsequently, these cells were cultured in the Roswell Park Memorial Institute-1640 medium supplemented with 10% fetal bovine serum (FBS; Gibco), 100 U/mL penicillin (FUJIFILM), 0.1 mg/mL streptomycin (FUJIFILM), 2 mM L-glutamine (Gibco), and 20 ng/mL macrophage colony-stimulating factor (M-CSF) (BioLegend) for 7 days to induce macrophage differentiation. BMDMs were polarized into M1 or M2 phenotype following treatment with 100 ng/mL LPS or 20 ng/mL IL-4, respectively. Specific incubation times for each experiment are specified in the figure legends.

### 2.5. TWNT-1 Human HSC Culture

TWNT-1 HSC cell line was purchased from the JCRB Cell Bank (JCRB1582) and cultured in the Dulbecco's modified Eagle's medium containing 10% FBS. To measure the activation of TWNT-1 cells into myofibroblasts, the cells were cultured in the BMDM-cultured medium for 48 h. The BMDM-cultured medium was a mixture of the culture medium collected 48 h after BMDM stimulation with or without IL-4 and an equal volume of Dulbecco's modified Eagle's medium containing 10% FBS. After culture for 48 h, RNA was extracted from BMDMs, and cDNA was synthesized for reverse transcription PCR (RT-PCR).

### 2.6. Reverse Transcription-Quantitative PCR (RT-qPCR)

Total RNA was extracted from cells using the RNeasy Micro Kit (Qiagen) and reverse-transcribed into cDNA using the GeneAce cDNA synthesis kit (NIPPON GENE). RT-PCR was performed using the THUNDERBIRD SYBR qPCR Mix (TOYOBO) with the 7500 Real-Time PCR System (Thermo Fisher Scientific). Gene expression levels were determined after normalization to the standard housekeeping gene, glyceraldehyde 3-phosphate dehydrogenase for mice or *18S rRNA* for humans, using the ΔCT method. All primers are listed in Table S2.

### 2.7. Immunohistochemistry

For immunohistochemistry, liver specimens were fixed with 4% paraformaldehyde (PFA) in 0.1 M phosphate buffer (pH 7.4), embedded in paraffin, and stained using the avidin–biotin complex (Vectastain Kit; Vector Laboratories). To detect hepatic fibrosis, the sections were further stained with Masson's trichrome. For immunofluorescence staining, liver specimens were fixed with 4% PFA and embedded in an OCT compound. The sections were stained with the primary antibodies listed in Table S1. Fluorescent-labeled secondary antibodies were used to detect the primary antibodies (Table S1). Nuclei were counterstained with 4′,6-diamidino-2-phenylindole (DAPI) (Life Technologies). Quantitative histological analysis was performed in a blinded manner.

### 2.8. Hydroxyproline Assay

Hydroxyproline content in the liver was measured using an assay kit (MAK008; Merck), following the manufacturer's instructions. An increase in the hydroxyproline content in the liver was set as the primary outcome.

### 2.9. Statistical Analyses

Data are represented as the mean ± standard error of the mean of at least three independent experiments. Statistical comparisons of means were performed using an unpaired two-tailed Student's *t*-test with the GraphPad Prism 8 software. Statistical significance was set at *p* < 0.05. For animal experiments, the following exclusion criteria were used: Abnormal health condition or death during the experiment, improper execution of the experiment (such as incorrect drug administration), outliers falling outside the range of mean ± 3 standard deviations, and technical failures.

## 3. Results

### 3.1. FABP7 in Macrophages Promotes Liver Fibrosis

**Figure 1:**
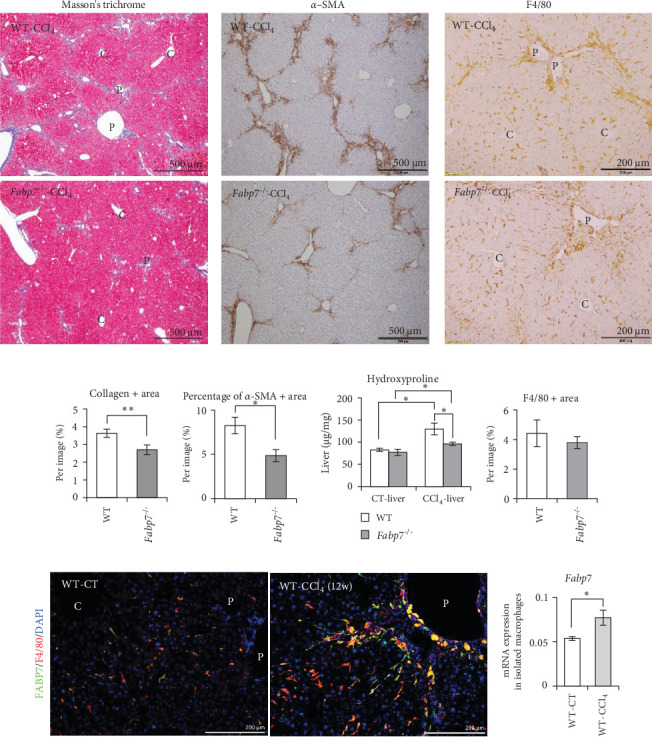
Loss of fatty acid-binding protein 7 (Fabp7) inhibits liver fibrosis progression. (A–C) Representative images of Masson's trichrome (MT) staining (A), *α*-smooth muscle actin (*α*-SMA) immunostaining (B), and F4/80 immunostaining (C) in the liver tissues of wild-type (WT) and *Fabp7*^−/−^ mice after carbon tetrachloride (CCl_4_) administration. (D) Percentage of MT (collagen)-positive areas in liver tissues (*n* = 5 or 6 mice/group). (E) Percentage of *α*-SMA-positive areas in liver tissues. (F) Hydroxyproline levels in WT and *Fabp7*^−/−^ liver tissues. (G) Percentage of F4/80-positive areas in liver tissues. (H) Representative images of fluorescent immunostaining (FABP7 [green]/F4/80 [red]/4′,6-diamidino−2-phenylindole [DAPI; blue]). (I) *Fabp7* mRNA levels in isolated hepatic macrophages of the WT-CT—and WT-CCl_4_-administered livers. Data are represented as the mean ± standard error (SE). *p* Values were determined via two-tailed Student's *t*-test (between two groups); *n* = 6 mice/group; *⁣*^*∗*^*p* < 0.05 and *⁣*^*∗∗*^*p*  < 0.01.

To clarify the role of FABP7 in hepatic macrophages, we established two distinct models: one by inducing hepatic fibrosis via long-term administration of CCl_4_ and the other by inducing MASH using a high-fat high-cholesterol (HFHC) diet. To induce liver fibrosis, CCl_4_ was intraperitoneally administrated to mice. Liver sections of *Fabp7*^−/−^ mice exhibited significantly decreased collagen levels compared to those of WT mice (Figure 1A,D). HSCs differentiate into myofibroblasts during hepatic fibrosis, characterized by the upregulation of *α*-smooth muscle actin (*α*-SMA) levels. Histological analysis comparing *α*-SMA levels in liver tissues revealed a significant reduction in *α*-SMA-positive regions in *Fabp7*^−/−^ mice compared to that in WT mice (Figure 1B,E). Additionally, hydroxyproline levels in the liver were lower in *Fabp7*^−/−^ mice than in WT mice (Figure 1F). Hepatic macrophage distribution in the liver of CCl_4_^−^administrated mice was increased in the periportal region (a highly fibrotic area). However, no significant difference in macrophage distribution in the fibrotic liver tissue was observed between WT and *Fabp7*^−/−^ mice (Figure 1C,G). Notably, FABP7 expression was enhanced in the macrophages clustered around the fibrotic area (Figure 1H). Consistent with the immunohistochemical results, mRNA levels of *Fabp7* in isolated hepatic macrophages were elevated in fibrotic livers compared to those in CT livers (Figure 1I).

To establish a MASH model, bone marrow chimeric mice were fed an HFHC diet (Figure S2A), considering the potential influence of brain-expressed FABP7 on HFHC diet intake [19]. These chimeric mice (*Fabp7*^−/−^ bone marrow transplantation [BMT] and WT BMT) were fed an HFHC or CT diet for 26 weeks to induce MASH. Initially, body weight, liver weight, white adipose tissue weight, and serum biochemical parameters were measured. Although the HFHC diet-fed mice showed signs of obesity and hepatic inflammation, no significant differences were observed between WT and *Fabp7*^−/−^ BMT mice (Figure S2B–D). Histological analysis confirmed FABP7 expression in hepatic macrophages after HFHC or CT diet. *Fabp7*^−/−^ BMT mice did not express FABP7 in almost all macrophages, indicating the replacement of recipient hepatic macrophages with *Fabp7*^−/−^ BMDMs (Figure S3A–D). Histological analysis of H&E-stained sections revealed severe hepatic steatosis, inflammation, and hepatocyte ballooning induced by the HFHC diet. However, no significant difference in the nonalcoholic fatty liver disease (NAFLD) activity scores (NAS) was observed between WT and *Fabp7*^−/−^ BMT mice (Figure S4A,B). Hepatic fibrosis was barely observed in the collagen immunostaining and hydroxyproline assays (Figure S4C–E).

These results suggest that macrophage Fabp7 promotes liver fibrosis but does not affect the extent of liver damage (rising NAS) caused by the HFHC diet.

### 3.2. Fabp7 Modulates Macrophage M2 Polarization

**Figure 2:**
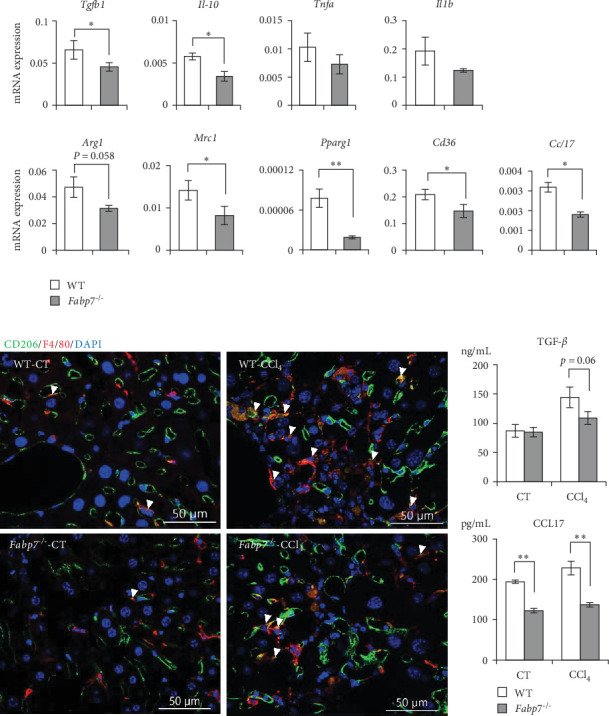
Fatty acid-binding protein 7 (Fabp7)-deficient hepatic macrophages inhibit M2 polarization during fibrosis. (A) mRNA expression levels of M1/M2-polarization-related genes in isolated hepatic macrophages of wild-type (WT)- and *Fabp7*^−/−^ carbon tetrachloride (CCl_4_)-administrated livers determined via quantitative reverse transcription-polymerase chain reaction (qRT-PCR). (B) Representative images of fluorescent immunostaining (CD206 [green]/F4/80 [red]/4′,6-diamidino-2-phenylindole [DAPI; blue]). (C) Levels of transforming growth factor (TGF)-*β* and CCL17 in the serum of WT and *Fabp7*^−/−^ mice with or without CCl_4_ administration determined via enzyme-linked immunosorbent assay (ELISA). Data are represented as the mean ± standard error (SE). *p* Values were determined via two-tailed Student's *t*-test (between two groups); *n* = 6 mice/group; *⁣*^*∗*^*p* < 0.05 and *⁣*^*∗∗*^*p* < 0.01.

Activated macrophages are involved in tissue fibrosis, especially pulmonary and renal fibrosis, in which M2-polarized macrophages promote fibrotic responses [20, 21]. However, the effect of M1/M2 macrophage polarization on the hepatic fibrotic response remains controversial. We investigated M1/M2 polarization and observed downregulation of M2-related gene (*Tgfb1*, *Il-10*, *Mrc1*, *Pparg1*, *Cd36*, and *Ccl17*) levels in *Fabp7*^−/−^ macrophages compared to those in WT macrophages. However, levels of M1-related genes (*Tnfa* and *Il-1 b*) were not significantly different between both cell types (Figure 2A). Levels of CD206 (Mrc1) in liver macrophages were observed via immunohistochemistry. CD206 expression was not prominent in the liver macrophages of CT livers (Figure 2B). Sinusoidal endothelial cells exhibited high CD206 levels. In CCl_4_-treated livers, CD206 expression was detected in the macrophages accumulated in the fibrotic regions. Consistent with the mRNA analysis results (Figure 2A), the number of CD206-expressing macrophages was lower in *Fabp7*^−/−^ mice than in WT mice (Figure 2B). Transforming growth factor-*β* (TGF-*β*) plays a crucial role in activating HSCs, thereby promoting liver fibrosis [22]. C–C motif chemokine ligand (CCL)-17 promotes pulmonary fibrosis [23]. Consequently, we measured the serum TGF-*β* and CCL17 concentrations in the CCl_4_-induced fibrosis model and CT mice (without CCl_4_). No significant difference in serum TGF-*β* levels was observed between WT mice and *Fabp7*^−/−^ mice in the fibrosis model, but a decreasing trend was observed in *Fabp7*^−/−^ mice. This was because serum TGF-*β* was produced by both hepatic macrophages and other cells, such as endothelial cells. Interestingly, serum CCL17 levels were lower in *Fabp7*^−/−^ mice than in WT mice in both the CT and fibrosis groups (Figure 2C). These findings suggest that FABP7 promotes the M2 polarization of hepatic macrophages, thereby leading to fibrosis.

### 3.3. BMDMs Express FABP7

**Figure 3:**
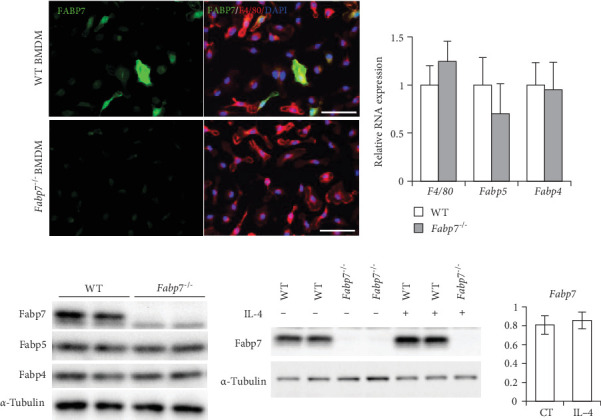
Bone marrow-derived macrophages (BMDMs) express fatty acid-binding protein 7 (Fabp7). (A) Representative images of FABP7 (green)/F4/80 (red)/4′,6-diamidino-2-phenylindole (DAPI; blue) staining in wild-type (WT) and *Fabp7*^−/−^ BMDMs. Scale bar, 50 μm. (B) Relative mRNA expression levels of *F4/80*, *Fabp4*, and *Fabp5* in WT and *Fabp7*^−/−^ BMDMs were determined via quantitative reverse transcription-polymerase chain reaction (qRT-PCR). (C) Protein expression levels of Fabps in WT and *Fabp7*^−/−^ BMDMs were determined via western blotting. (D) Protein expression levels of Fabp7 in WT and *Fabp7*^−/−^ BMDMs with or without interleukin (IL)-4 stimulation determined via western blotting. *p* Values were determined via a two-tailed Student's *t*-test (between two groups); *n* = 3 mice/group.

To clarify the role of FABP7 in macrophage polarization, experiments were performed using BMDMs of *Fabp7*^−/−^ and WT mice. First, we investigated the expression levels of FABPs in BMDMs. Immunocytochemistry revealed that ~40% of WT-BMDMs expressed FABP7 (Figure 3A). Levels of FABP4 and FABP5, which are expressed in BMDMs [13, 24], in WT and *Fabp7*^−/−^ BMDMs were also assessed via qPCR and western blotting. No significant differences in the expression levels of FABP4 and FABP5 were observed between WT and *Fabp7*^−/−^-BMDMs (Figure 3B,C). Furthermore, no difference in FABP7 levels was observed between WT BMDMs with and without IL-4 stimulation (Figure 3D).

### 3.4. FABP7 in BMDMs Is Involved in Anti-Inflammatory Polarization

**Figure 4:**
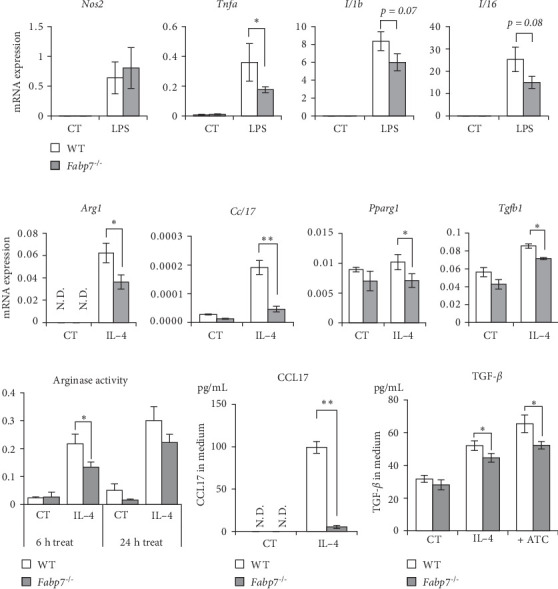
Fatty acid-binding protein 7 (Fabp7) in bone marrow-derived macrophages (BMDMs) is involved in macrophage M2 polarization. (A) mRNA expression levels of M1 polarization-related genes in wild-type (WT) and *Fabp7* BMDMs with or without lipopolysaccharide (LPS) treatment (10 ng/mL; 6 h) determined via quantitative reverse transcription-polymerase chain reaction (qRT-PCR). (B) mRNA expression levels of M2-polarization-related genes in WT and *Fabp7*^−/−^ BMDMs with or without interleukin (IL)-4 treatment (20 ng/mL; 6 h) determined via qRT-PCR. (C) Arginase activities in WT and *Fabp7*^−/−^ BMDMs with or without IL-4 (20 ng/mL; 6 or 24 h) treatment were determined using assay kits. (D) CCL17 levels in the culture media of WT- and *Fabp7*^−/−^- BMDMs with or without IL-4 treatment (20 ng/mL; 48 h) treatment determined via enzyme-linked immunosorbent assay (ELISA). (E) Transforming growth factor (TGF)-*β* levels in the cultured media of WT and *Fabp7*^−/−^ BMDMs with or without IL-4 (20 ng/mL; 48 h) and apoptotic thymocyte (ATC) treatment determined via ELISA. Data are represented as the mean ± standard error (SE). *p* Values were determined via two-tailed Student's *t*-test (between two groups); *n* = 4–6 mice/group; *⁣*^*∗*^*p* < 0.05, *⁣*^*∗∗*^*p* < 0.01, and *⁣*^*∗∗∗*^*p* < 0.001.

Next, BMDMs (WT vs. *Fabp7*^−/−^) were polarized into M1 and M2 macrophages using LPS and IL-4, respectively. mRNA levels of M1/M2-related genes were determined via RT-PCR. After LPS treatment, no significant difference in *Nos2* levels was observed between *Fabp7*^−/−^ and WT mice. *Tnfa* levels were lower in *Fabp7*^−/−^ mice than in WT mice. *Il1b* and *Il6* levels were also downregulated, but the difference between both groups was not statistically significant (Figure 4A). After IL-4 treatment, mRNA levels of major M2-related genes (*Arg1*, *Ccl17*, *Tgfb1*, and *Pparg1*) were lower in *Fabp7*^−/−^ BMDMs than in WT BMDMs (Figure 4B). Based on these results and mRNA expression data of hepatic macrophages isolated from fibrotic livers (Figure 2A), we further investigated the involvement of Fabp7 in macrophage M2 polarization. Arginase activity was diminished in *Fabp7*^−/−^ BMDMs compared to that in WT BMDMs (Figure 4C). Subsequently, production of CCL17 and TGF-*β* was confirmed via enzyme-linked immunosorbent assay. Upon IL-4 stimulation or coculture with apoptotic thymocytes (ATCs), levels of CCL17 and TGF-*β* in the culture medium were significantly lower in *Fabp7*^−/−^ BMDMs than in WT BMDMs (Figure 4D,E). These results suggest that FABP7 promotes IL-4-induced M2 polarization of macrophages.

### 3.5. Fabp7 Regulates M2-Related Genes via Peroxisome Proliferator-Activated Receptor (PPAR)-*γ* Expression and Activity

**Figure 5:**
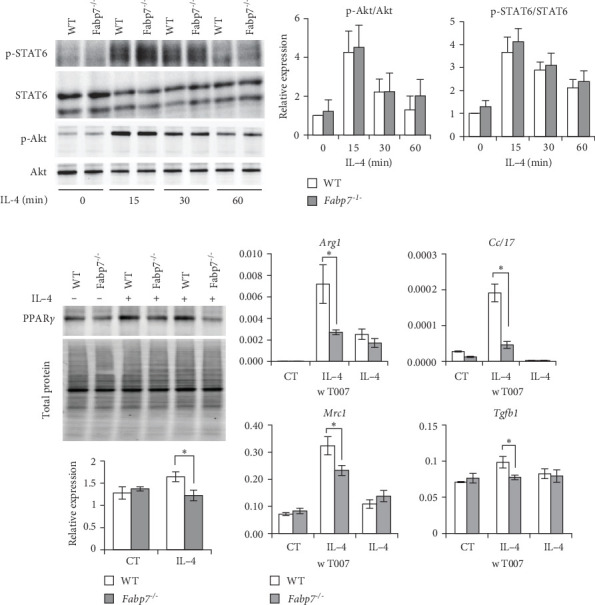
Fatty acid-binding protein 7 (Fabp7) in macrophages modulates M2 polarization via peroxisome proliferator-activated receptor (PPAR)-*γ* expression. (A) Protein expression/phosphorylation levels of signal transducer and activator of transcription 6 (STAT6) and Akt in wild-type (WT) and *Fabp7*^−/−^ bone marrow-derived macrophages (BMDMs) with or without interleukin (IL)-4 treatment (20 ng/mL; indicated time points) determined via western blotting. (B) Relative phosphorylation levels of STAT6 and Akt were analyzed using the Image Lab software. (C) Protein expression levels of PPAR*γ* in WT and *Fabp7*^−/−^ BMDMs with or without IL-4 treatment (20 ng/mL; 12 h) were determined via western blotting. (D) mRNA expression levels of M2 polarization-related genes in WT and *Fabp7*^−/−^ BMDMs with or without IL-4 (20 ng/mL; 6 h) or T0070907, a PPAR*γ* selective antagonist, treatment (10 mM) determined via quantitative reverse transcription-polymerase chain reaction (qRT-PCR). Data are represented as the mean ± standard error (SE). *p* Values were determined via two-tailed Student's *t*-test (between two groups); *n* = 4 mice/group; *⁣*^*∗*^*p* < 0.05, *⁣*^*∗∗*^*p* < 0.01, and *⁣*^*∗∗∗*^*p* < 0.001.

STAT6 and Akt signaling pathways are activated (phosphorylated) by IL-4 stimulation, promoting M2 polarization of macrophages [25]. Therefore, we compared the phosphorylation levels of STAT6 and Akt in WT and *Fabp7*^−/−^ BMDMs after IL-4 treatment. Notably, no significant differences in phosphorylation levels were observed between WT and *Fabp7*^−/−^ BMDMs (Figure 5A,B).

PPAR*γ* is a marker protein for M2 polarization and transcription factor regulating the expression of various M2-related genes (e.g., *Arg1* and *Mrc1*) [26]. PPAR*γ* levels following IL-4 stimulation were lower in *Fabp7*^−/−^ BMDMs than in WT BMDMs (Figure 5C). Prior to the induction of M2 polarization by IL-4 stimulation, treatment with T0070907, a PPAR*γ* inhibitor, suppressed M2-related gene expression. *Arg1*, C*cl17*, *Mrc1*, and *Tgfb1* levels were not significantly different between WT and *Fabp7*^−/−^ BMDMs after T0070907 treatment, suggesting that these genes were regulated in a PPAR-dependent manner (Figure 5D).

These results suggest that FABP7 plays a key role in macrophage M2 polarization by regulating PPAR*γ* expression.

### 3.6. *FABP7* in Hepatic Macrophages Modulates Myofibroblast Activation and CD4^+^ T-Cell Infiltration by Regulating Macrophage M2 Polarization

**Figure 6:**
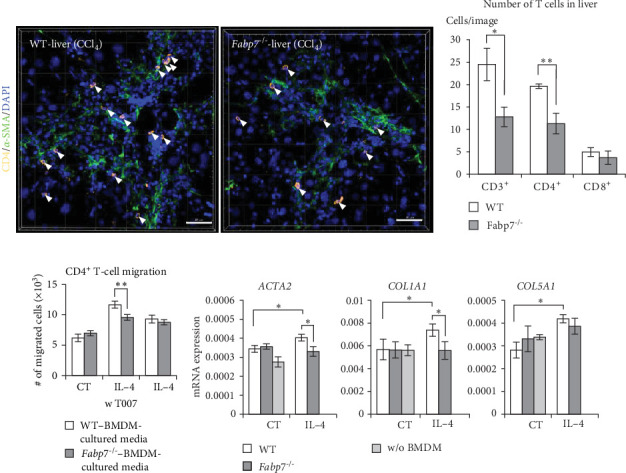
Fatty acid-binding protein 7 (Fabp7) in macrophages promotes CD4^+^ T-cell migration and fibroblast activation via M2 polarization. (A) Representative images of CD4 (yellow)/*α*-smooth muscle actin (*α*-SMA) (green)/4′,6-diamidino-2-phenylindole (DAPI; blue) staining in liver tissues of wild-type (WT) and *Fabp7*^−/−^ carbon tetrachloride (CCl_4_)-administered mice. Scale bar, 50 μm. (B) Numbers of CD3^+^, CD4^+^, and CD8^+^ cells in liver tissues of W and *Fabp7*^−/−^ mice after CCl_4_ administration. (C) Numbers of migrated CD4^+^ thymocytes into the chamber of bone marrow-derived macrophage (BMDM)-culture medium. (D) mRNA expression levels of actin alpha 2 (*ACTA2*), collagen (*COL*)-*1A1*, and *COL5A1* in TWNT-1 cells after culturing in WT- and *Fabp7*^−/−^-BMDM cultured media determined via quantitative reverse transcription-polymerase chain reaction (qRT-PCR). Data are represented as the mean ± standard error (SE). *p* Values were determined via two-tailed Student's *t*-test (between two groups); *n* = 6 mice/group; *⁣*^*∗*^*p* < 0.05 and *⁣*^*∗∗*^*p* < 0.01.

Tissue macrophages play a role in regulating T-cell migration. CCL17 induces the migration of helper T (Th) cells, along with C–C motif chemokine receptor (CCR)-4 (receptor for CCL17), which is strongly expressed in Th cells [21]. Th cells promote fibrotic responses [27, 28]. Therefore, we compared the migration of Th (CD4^+^ T), CD3^+^ T, and CD8^+^ cytotoxic T cells into the liver in the CCl_4_-induced fibrosis model. CD4^+^ cell migration was observed around the fibrotic areas of the liver (Figure 6A). Measurement of T-cell migration per unit area of liver tissue revealed significantly lower migration of CD3^+^ and CD4^+^ T cells in *Fabp7*^−/−^ mice than in WT mice (Figure 6B). The number of CD8^+^ T cells was lower than that of CD4^+^ cells in the livers of both groups and did not differ significantly between WT and *Fabp7*^−/−^livers (Figure 6B).

CD4^+^ cells isolated from the thymus of WT mice expressing CCR4 (Figure S5) exhibited enhanced migration in the IL-4-stimulated BMDM-cultured medium compared to that in the CT BMDM-cultured medium. However, migration of CD4^+^ T cells in the IL-4-stimulated *Fabp7*^−/−^ BMDM-cultured medium was significantly lower than that in the IL-4-stimulated WT BMDM-cultured medium (Figure 6C). These differences were counteracted by T0070907 when BMDMs were stimulated with IL-4. This suggests that FABP7 is involved in CD4^+^ T-cell migration by regulating M2 polarization via PPAR*γ* activation.

The effect of M2-polarized macrophages on fibroblast response was also investigated. A human HSC line (TWNT-1) was cultured in a BMDM-cultured medium with or without IL-4 stimulation. *ACTA2*, collagen type I alpha 1 (*COL1A1*), and *COL5A1* levels as markers of stellate cell activation and differentiation into myofibroblasts in TWNT-1 cells were determined via qRT-PCR after 48 h of culture in the BMDM-cultured medium. Levels of these mRNA in TWNT-1 cells were elevated in the IL-4-treated BMDM-cultured medium compared to those in the CT BMDM-cultured medium. Moreover, mRNA levels of *ACTA2* and *COL1A1* in TWNT-1 cells cultured in IL-4-stimulated *Fabp7*^−/−^ BMDM-cultured medium were lower than those in the IL-4-stimulated WT-BMDM cultured medium (Figure 6D). This result suggests that the impairment of M2 polarization due to FABP7 deficiency in macrophages suppresses the fibrotic responses in TWNT-1 cells.

In summary, FABP7 in hepatic macrophages promotes liver fibrosis by regulating the migration of Th cells and the fibrotic response of HSCs via modulation of macrophage M2 polarization.

## 4. Discussion

In this study, we demonstrated that FABP7 in hepatic macrophages regulated M2 polarization and promoted liver fibrosis by activating fibroblasts and regulating CD4^+^ T-cell migration. The fibrotic response has a beneficial effect by suppressing the spread of inflammation and tissue damage and promoting tissue repair. However, an excessive fibrotic response also has a detrimental effect by increasing the risk of cirrhosis and hepatocellular carcinoma. Hence, controlling the fibrotic response at the appropriate time and extent is important for tissue regeneration. Macrophages play a central role in tissue injury and repair by polarizing to M1/M2. Our study revealed that FABP7 in hepatic macrophages enhances liver fibrosis by regulating M2 polarization. Therefore, FABP7 in hepatic macrophages may be a potential therapeutic target for liver fibrosis.

M2 macrophages typically contribute to tissue repair by producing anti-inflammatory cytokines and phagocytosing the dead cells. TGF-*β* and CCL17, upregulated in M2 macrophages, are recognized for their stimulation of ECM production by fibroblasts, leading to fibrosis [1, 29]. Furthermore, CCR4, a receptor for CCL17, is expressed in Th cells. Recent findings suggest that CCL17 governs Th-cell activation, including the production of IL-4 and IL-13. The diminished expression of TGF-*β* and CCL17 in *Fabp7*^−/−^ macrophages may attenuate liver fibrosis by reducing myofibroblast activation and the migration of Th cells into the liver tissue.

PPAR*γ* serves as a critical transcription factor regulating intracellular lipid metabolism and controlling the expression of M2-related genes [30]. Studies have shown that M2-polarized alveolar macrophages exacerbate pulmonary fibrosis through PPAR*γ* activation [31]. Our current research revealed a reduction of PPAR*γ* expression in *Fabp7*^−/−^ macrophages after IL-4 stimulation. Moreover, treatment with a PPAR*γ* inhibitor resulted in the reduced expression of several M2-related genes. These findings suggest that FABP7 modulates M2 polarization by influencing PPAR*γ* expression. While STAT6 serves as a major transcription factor responsible for upregulating PPAR*γ* in macrophages [32, 33], our study shows that FABP7 deficiency has no impact on STAT6 phosphorylation levels, indicating that FABP7 regulates PPAR*γ* expression through an alternative pathway.

Transcription factor GATA-binding protein 3 (GATA3) is a critical regulator of both innate and adaptive immunity. GATA3 expression is regulated by various signaling pathways, including the IL-4-STAT6, mTOR, and Notch pathways, which are essential for the differentiation and functional regulation of T cells [34]. It is transiently expressed in macrophages in response to IL-4, which CTs the expression of Arg-1 [35], suggesting that GATA3 is an important transcription factor involved in the regulation of macrophage polarization. However, its interaction with FABP molecules remains unclear, warranting further investigation.

Recent studies have highlighted epigenetic regulation as a potential mechanism underlying macrophage polarization [36]. Histone deacetylase inhibitors suppress the expression of PPAR*γ* and M2 polarization after IL-4 stimulation [25]. We recently reported that FABP7 in astrocytes interacts with ATP citrate lyase in the nucleus, participating in histone acetylation [37] and that FABP7 binding to oleic acid (OA) in glioma cells modulates histone acetylation [38]. Epigenetic regulation is influenced by diverse environmental factors and possibly contributes to organ-specific functions of macrophages. However, further studies are necessary to elucidate the roles of FABP7 in the epigenetic regulation of macrophage polarization.

Omega-3 polyunsaturated fatty acids (PUFAs) such as docosahexaenoic acid (DHA), eicosapentaenoic acid (EPA), and *α*-linolenic acid (ALA) have been reported to induce M2 macrophages or an anti-inflammatory phenotype [39]. OA (omega-9 monounsaturated FA) treatment induces M2-like macrophages by promoting lipid droplet formation and mitochondrial FA oxidation [40]. Although FA and lipid metabolism are involved in the regulation of macrophage M2-polarization, the precise molecular mechanisms remain unknown. FABP7 has a high binding affinity for omega-3 PUFAs and OA [41]. We have previously reported that FABP7 in astrocytes participates in omega-3 PUFA (ALA) uptake and lipid droplet formation [42, 43]. Thus, FABP7 may regulate the interaction between fatty acid metabolism and macrophage polarization.

Deletion/inhibition of FABP5 in macrophages enhances M2 polarization [44]. Hou et al. [14] reported that unsaturated fatty acids, such as OA, accumulate in FABP5-deficient macrophages, activate PPAP*γ* signaling, and promote M2 polarization. In contrast, our study revealed that FABP7-deficient macrophages impaired PPAR*γ* expression and M2 polarization after IL-4 stimulation. The distinct binding affinities of FABP5 and FABP7 for fatty acids [41, 45], along with the diverse expression patterns of FABP subtypes in macrophages, possibly influence PPAR*γ* function and M2 polarization by modulating the intracellular fatty acid composition and metabolism. However, regulatory mechanisms underlying FABP expression in macrophages remain unclear. The diversity of FABP expression patterns in macrophages possibly confer functions suitable for the specific microenvironment.

## 5. Conclusions

Overall, this study showed that FABP7 in hepatic macrophages regulated PPAR*γ* expression and M2 polarization, thereby promoting liver fibrosis via fibroblast activation and CD4^+^ T-cell migration. Therefore, regulation of hepatic macrophage function by modulating FABP7 expression and ligand binding is a promising target to treat liver diseases and maintain liver homeostasis.

## Data Availability

The data supporting the findings of this study are available upon request from the corresponding author.

## Nomenclature

Akt: protein kinase B
ALA: alpha-linolenic acid
ALT: alanine aminotransferase
AST: aspartate aminotransferase
*α*-SMA: *α*-smooth muscle actin
ATC: apoptotic thymocyte
BMDM: bone marrow-derived macrophage
BMT: bone marrow transplantation
CCl_4_: carbon tetrachloride
CD: cluster of differentiation
DAPI: 4′,6-diamidino-2-phenylindole
DHA: docosahexaenoic acid
ECM: extracellular matrix
EPA: eicosapentaenoic acid
FABP: fatty acid-binding protein
HFHC: high-fat high-cholesterol
HSC: hepatic stellate cell
IL: interleukin
KC: Kupffer cell
LPS: lipopolysaccharides
NAFLD: nonalcoholic fatty liver disease
NASH: nonalcoholic steatohepatitis
MASH: metabolic dysfunction-associated steatohepatitis
M-CSF: macrophage colony-stimulating factor
PFA: paraformaldehyde
PPAR: peroxisome proliferator-activated receptor
PUFA: polyunsaturated fatty acid
STAT6: signal transducer and activator of transcription 6
TGF-*β*: transforming growth factor-*β*.

## Supporting Information

Supporting Information Table S1. List of antibodies used for western blotting, immunohistochemistry, and flow cytometry; Table S2. List of primers used for reverse transcription-quantitative polymerase chain reaction (RT-qPCR); Figure S1. Fluorescence-activated cell sorting (FACS) for the isolation of hepatic macrophages. (A) Procedure for gating single and live cell populations in the liver tissues. (B) Gating of CD45+ cells (myeloid cells) in liver cells. (C) Representative images of CD11b+/F4/80+/Mer+ cells isolated from hepatic macrophages; Figure S2. Metabolic dysfunction-associated steatohepatitis (MASH) induced by high-fat high-cholesterol (HFHC) diet after bone marrow transplantation (BMT). (A) Procedure for establishing a MASH model post-BMT. For further details, please refer to the Supporting Information and Section 2. (B) Body weights of BMT mice in the control and HFHC diet groups (*n* = 8/group). (C) Liver and epididymal white adipose tissue (eWAT) weights of BMT mice after intake of control or HFHC diet for 26 weeks (*n* = 8/group). (D) Results of blood tests of BMT mice after intake of control or HFHC diet for 26 weeks (*n* = 8/group); Figure S3. Hepatic macrophages do not express Fabp7 after *Fabp7*^−/−^-BMT. (A) Representative images of the fluorescent immunostaining (F4/80 [red]/FABP7 [green]/4“,6-diamidino−2-phenylindole [DAPI; blue]) of the liver tissues of wild-type (WT) and *Fabp7*^−/−^-BMT mice after 26 weeks of control diet. (B) Percentage of Fabp7+ F4/80+ cells in liver tissues (*n* = 4/group). (C) Representative images of the fluorescent immunostaining (F4/80 [red]/FABP7 [green]/DAPI [blue]) of the liver tissues of WT- and *Fabp7*^−/−^-BMT mice after 26 weeks of HFHC diet. (D) Percentage of Fabp7+ cells among F4/80+ cells in liver tissues (*n* = 6/group); Figure S4. Fabp7 in hepatic macrophages does not affect liver damage induced by HFHC diet. (A) Representative images of the hematoxylin and eosin (H&E) staining of liver tissues of WT- and *Fabp7*^−/−^-BMT mice after HFHC diet intake for 26 weeks. (B) NAFLD activity score (NAS) in the liver tissues of WT- and *Fabp7*^−/−^ BMT mice after HFHC diet intake for 26 weeks determined via histological assessment (*n* = 8/group). (C) Representative images of collagen 1 immunostaining in the liver tissues of WT- and *Fabp7*^−/−^-BMT mice after HFHC diet intake for 26 weeks. (D) Percentage of collagen 1-positive areas in the liver tissues of WT- and *Fabp7*^−/−^-BMT mice after HFHC diet intake for 26 weeks (*n* = 8/group). (E) Hydroxyproline content in the liver tissues of WT- and *Fabp7*^−/−^-BMT mice after control HFHC diet intake for 26 weeks (*n* = 4−8/group); Figure S5. C−C motif chemokine receptor 4 (CCR4) expression levels in T cells isolated from the thymus and spleen. (A) CD4 and CD8 expression levels in thymocytes after the elimination of dead or doublet cells. (B) CCR4 expression levels in CD4^+^ and CD4^+^ CD8^+^ cells. (C) CD4 and CD8 expression levels in splenocytes after the elimination of dead or doublet cells. (D) CCR4 expression levels in CD4^+^ and CD8^+^ cells.
