# Supplementary material for: FABP7 in Hepatic Macrophages Promotes Fibroblast Activation and CD4+ T-Cell Migration by Regulating M2 Polarization During Liver Fibrosis
Source: J Immunol Res. 2025 Feb 19;2025:6987981. doi: 10.1155/jimr/6987981 (PMC11865460; doi:10.1155/jimr/6987981)
Supplement: Supporting Information — Table S1. List of antibodies used for western blotting, immunohistochemistry, and flow cytometry; Table S2. List of primers used for reverse transcription-quantitative polymerase chain reaction (RT-qPCR); Figure S1. Fluorescence-activated cell sorting (FACS) for the isolation of hepatic macrophages. (A) Procedure for gating single and live cell populations in the liver tissues. (B) Gating of CD45+ cells (myeloid cells) in liver cells. (C) Representative images of CD11b+/F4/80+/Mer+ cells isolated from hepatic macrophages; Figure S2. Metabolic dysfunction-associated steatohepatitis (MASH) induced by high-fat high-cholesterol (HFHC) diet after bone marrow transplantation (BMT). (A) Procedure for establishing a MASH model post-BMT. For further details, please refer to the Supporting Information and Section 2. (B) Body weights of BMT mice in the control and HFHC diet groups (n = 8/group). (C) Liver and epididymal white adipose tissue (eWAT) weights of BMT mice after intake of control or HFHC diet for 26 weeks (n = 8/group). (D) Results of blood tests of BMT mice after intake of control or HFHC diet for 26 weeks (n = 8/group); Figure S3. Hepatic macrophages do not express Fabp7 after Fabp7−/−-BMT. (A) Representative images of the fluorescent immunostaining (F4/80 [red]/FABP7 [green]/4“,6-diamidino−2-phenylindole [DAPI; blue]) of the liver tissues of wild-type (WT) and Fabp7−/−-BMT mice after 26 weeks of control diet. (B) Percentage of Fabp7+ F4/80+ cells in liver tissues (n = 4/group). (C) Representative images of the fluorescent immunostaining (F4/80 [red]/FABP7 [green]/DAPI [blue]) of the liver tissues of WT- and Fabp7−/−-BMT mice after 26 weeks of HFHC diet. (D) Percentage of Fabp7+ cells among F4/80+ cells in liver tissues (n = 6/group); Figure S4. Fabp7 in hepatic macrophages does not affect liver damage induced by HFHC diet. (A) Representative images of the hematoxylin and eosin (H&E) staining of liver tissues of WT- and Fabp7−/−-BMT mice after HFHC diet intake for [file 6987981.f1.pdf]

Supplementary Description

1. Materials and Methods

1.1. Mouse Bone Marrow Transplantation and HFHC diet feeding study

Bone marrow transplantation (BMT) experiments were performed according to previously reported methods with some modifications [1,2]. Bone marrow cells were collected from the tibias and femurs of 6- to 8- week-old wild-type (WT) and *Fabp7*$^{-/-}$ male mice by rinsing. Mononuclear cells were isolated from the bone marrow using density centrifugation on Histopaque-1083 (Sigma-Aldrich, Co.). The recipient 7-week-old WT mice were lethally irradiated with 9 Gy using a γ-ray machine (Hitachi Medical Co.) and subsequently received 1 x 10^7 donor bone marrow mononuclear cells intravenously via the tail vein. To confirm successful BMT, genotyping PCR was performed on DNA collected from blood and tail tissue at 2 weeks post-BMT. At 2 weeks post-BMT, 10 mg/kg body weight of clodronate liposomes (Hygieia Bioscience) were administered intraperitoneally to replace resident liver macrophages with transplanted bone marrow cell-derived macrophages. Two weeks after clodronate liposome administration, mice were fed a high-fat, high-cholesterol diet (HFHC diet, D09100310, Research Diet) or a normal diet for 26 weeks to induce metabolic dysfunction-associated steatohepatitis (MASH). Body weight and food consumption were monitored twice a

week.

1. 2. Assessment of NAFLD activity score (NAS)

MASH was assessed using the non-alcoholic fatty liver disease (NAFLD) activity score (NAS) on hematoxylin and eosin (H&E) stained liver sections of according to a previous report [3]. The total NAS is a cumulative score that includes the assessment of steatosis (0-3), lobular inflammation (0-3) and ballooning (0-2) and ranging from 0 to 8. The diagnosis of MASH or, conversely, differentiation from fatty liver not indicative of MASH is usually made first. The NAS is then used to grade the level of activity observed in the liver. In the reference literature, 1) NAS scores of 0 to 2 are generally observed in cases that are not considered indicative of MASH; 2) scores of 3 to 4 are evenly distributed among cases that are not indicative, borderline, or considered MASH; 3) scores of 5 to 8 are more likely to be found in cases that are considered MASH.

1. 3. Isolation and culture of thymocytes

Thymus and spleen tissues were harvested from 7-week-old WT mice and mechanically dispersed by mashing through a 40 μm mesh using a 1 mL injection wick. The cell suspension was stained with the fluorescence-labelled antibodies and then analyzed by

flow cytometry. The antibodies used for staining are listed in Table S1. Flow cytometric analysis revealed that CD4-positive cells in the thymus expressed CCR4, whereas CD4-positive cells in the spleen did not express this marker (Figure S5A-D). Following this analysis, thymocytes were stained with PE-CD4 antibody, reacted with anti-PE microbeads and isolated using the Magnetic-Activated Cell Sorting (MACS) technique. The isolated CD4-positive thymocytes were then cultured in RPMI1640 medium supplemented with anti-CD3 (2 μg/mL, BD Biosciences, 553058) and anti-CD28 (5 μg/mL, BD Biosciences, 553295) antibodies, together with 10% FBS, 2 mM L-glutamine, 1x penicillin-streptomycin (P/S), and 2 mM β-mercaptoethanol. These cells were used for the subsequent experiments.

1. 4. Transwell migration assay

T-cell migration was assessed using 3-μm pore 24-transwell plates (Corning Life Sciences) following previously reported protocols with some modifications [4,5]. The lower chamber of the transwell plate was filled with the supernatant obtained from the BMDM cultures. To prepare the supernatant, BMDMs (both WT and *Fabp7*$^{-/-}$) were cultured for 48 h with or without IL-4 (20 ng/mL) and T0070907 (20 μM, a PPARγ specific antagonist, Selleck). The supernatant was collected, centrifuged, and combined

with an equal volume of fresh T cell culture medium to give a 50% mixed medium. The combined medium was then added to the bottom chamber of the transwell plate. Isolated $CD4^+$ thymocytes (4 x 10^5 cells 0.1 mL) were added to the upper chamber. After 24 hours of T-cell placement in the upper chamber, the number of T-cell infiltrating the lower chamber was counted.

1. 5. ELISA

The concentrations of TGF-β (R&D Systems, DB100C) and CCL17 (R&D Systems, MCC170) in BMDM culture media and mouse serum were measured using an ELISA kit according to the manufacturer's instructions.

1. 6. Western blot

BMDMs and 3T3 cells were extracted after stimulation with RIPA buffer (Thermo Scientific) containing protease and phosphatase inhibitor cocktails (Roche). Protein concentrations were determined using the BCA protein assay kit (Thermo Fisher Scientific). Lysates were mixed with SDS-PAGE loading buffer, boiled at 100 ℃ for 5 min, and then subjected to 10% or 12% SDS-PAGE. Proteins were transferred to PVDF membranes (Millipore). The membranes were blocked for 1 hour in TBS-T containing

5% bovine serum albumin. They were subsequently incubated overnight at 4 ℃ with primary antibodies, as detailed in Table S1. After washing, the membranes were incubated with peroxide-conjugated secondary antibodies (Table S1) for 1 hour at room temperature. Protein expression was detected by chemiluminescence, and protein signal levels were quantified using Image Lab software (Bio-Rad).

1. 7. Arginase activity assay

Arginase activity in BMDMs was measured using an arginase activity assay kit (Merck, MAK112) according to the manufacturer's instructions.

Table S1. List of antibodies used for western blotting, immunohistochemistry, and flow cytometry

Primary antibodies for western blotting

anti-mouse FABP4 Antibody	Cell Signaling Technology	Cat# 2120
anti-mouse FABP5 (D1A7T) Antibody	Cell Signaling Technology	Cat# 39926
anti-mouse FABP7 Antibody	Owada, 2008	N/A
anti-mouse α -Tubulin (YL1/2) Antibody	Santa Cruz	Cat# sc-53029

anti-human/mouse Fibronectin Antibody	abcam	Cat# ab2413
anti-mouse α-SMA (D4K9N) Antibody	Cell Signaling Technology	Cat# 19245
anti-mouse β-actin Antibody	Santa Cruz	Cat# sc-47778
anti-human/mouse PPARγ (D69) Antibody	Cell Signaling Technology	Cat# 2430
anti-mouse pSTAT6 (phospho Y641) Antibody	abcam	Cat# ab28829
anti-mouse STAT6 Antibody	Santa Cruz	Cat# sc-374021
anti-mouse Phospho-Akt (Ser473) (D9E)Antibody	Cell Signaling Technology	Cat# 4060
anti-human/mouse Akt Antibody	Cell Signaling Technology	Cat# 9272
**Secondary antibodies for western blotting**		
HRP anti-rabbit IgG Antibody	Merck	Cat# AP307P
HRP anti-rat IgG Antibody	Merck	Cat# AP136P
**Primary antibodies for immunohistochemistry**		
anti-mouse F4/80 (CI:A3-1) Antibody	BIO RAD	Cat# MCA497GA
anti-mouse Fabp7 Antibody	Owada, 2008	N/A
anti-mouse α-SMA (D4K9N) Antibody	Cell Signaling Technology	Cat# 19245
anti-mouse CD3 (17A2) Antibody	BioLegend	Cat# 100201
anti-mouse CD4 (RM4-5) Antibody	BioLegend	Cat# 100505

anti-mouse CD8 (YTS156.7.7) Antibody	BioLegend	Cat# 126602
**Secondary antibodies for immunohistochemistry**		
AlexaFlour488 anti-rabbit IgG Antibody	ThermoFisher Scientific	Cat# A11070
AlexaFlour568 anti-rat IgG Antibody	ThermoFisher Scientific	Cat# A11077
**Antibodies for flow cytometry**		
FITC anti-mouseCD45 (30-F11) Antibody	BioLegend	Cat# 103107
PE anti-mouse/human CD11b (M1/70) Antibody	BioLegend	Cat# 101208
BV421 anti-mouseF4/80 (BM8) Antibody	BioLegend	Cat# 123132
APC anti-mouse Mer (2B10C42) Antibody	BioLegend	Cat# 151507
PE anti-mouse CD4 Antibody	BioLegend	Cat# 100407
BV421 anti-mouse CD3 Antibody	BioLegend	Cat# 100227
PerCP/Cy5.5 anti-mouse CD8 Antibody	BioLegend	Cat# 140417
APC anti-mouse CD194 (CCR4) Antibody	BioLegend	Cat# 131211

Table S2. List of primers used for reverse transcription-quantitative polymerase chain reaction (RT-qPCR)

Name	**5' - 3'**
mGapdh-Fw	AGGTCGGTGTGAACGGATTTG
mGapdh-Rv	GGGGTCGTTGATGGCAACA
mFabp4-Fw	AAGGTGAAGAGCATCATAACCCT
mFabp4-Rv	TCACGCCTTTCATAACACATTCC
mFabp5-Fw	TGAAAGAGCTAGGAGTAGGACTG
mFabp5-Rv	CTCTCGGTTTTGACCGTGATG
mFabp7-F	AAGTGGGAAACGTGACCAAAC
mFabp7-R	CAACCGAACCACAGACTTACAG
mTgfb1-Fw	CTCCCGTGGCTTCTAGTGC
mTgfb1-Rv	GCCTTAGTTTGGACAGGATCTG
mIl10-Fw	GCTCTTACTGACTGGCATGAG
mIl10-Rv	CGCAGCTCTAGGAGCATGTG
mTnfa-Fw	CCCTCACACTCAGATCATCTTCT
mTnf-Rv	GCTACGACGTGGGCTACAG
mIl1b-Fw	GCAACTGTTCCTGAACTCAACT

mIl1b-Rv	ATCTTTTGGGGTCCGTCAACT
mNos2-Fw	GTTCTCAGCCCAACAATACAAGA
mNos2-Rv	GTGGACGGGTCGATGTCAC
Il6-Fw	TCTATACCACTTCACAAGTCGGA
Il6-Rv	GAATTGCCATTGCACAACTCTTT
mArg1-Fw	CTCCAAGCCAAAGTCCTTAGAG
mArg1-Rv	AGGAGCTGTCATTAGGGACATC
mMrc1-Fw	CTCTGTTCAGCTATTGGACGC
mMrc1-Rv	CGGAATTTCTGGGATTCAGCTTC
mPparg1-Rv	ATGGCATTGTGAGACATCCCC
mPparg1-F	CTCCAAGAATACCAAAGTGCGA
mCd36-Fw	ATGGGCTGTGATCGGAACTG
mCd36-Rv	TTTGCCACGTCATCTGGGTTT
mCcl17-Fw	AGTGGAGTGTTCCAGGGATG
mCcl17-Rv	CCAATCTGATGGCCTTCTTC
h18SrRNA-Fw	CTACCACATCCAAGGAAGCA
h18SrRNA-Rv	TTTCGTCACTACCTCCCCG
hACTA2-Fw	CTATGAGGGCTATGCCTTGCC

hACTA2-Rv	GCTCAGCAGTAGTAACGAAGGA
hCOL1A1-Fw	GAGGGCCAAGACGAAGACATC
hCOL1A1-Rv	CAGATCACGTCATCGCACAAC
hCOL5A1-Fw	TACAACGAGCAGGGTATCCAG
hCOL5A1-Rv	ACTTGCCATCTGACAGGTTGA

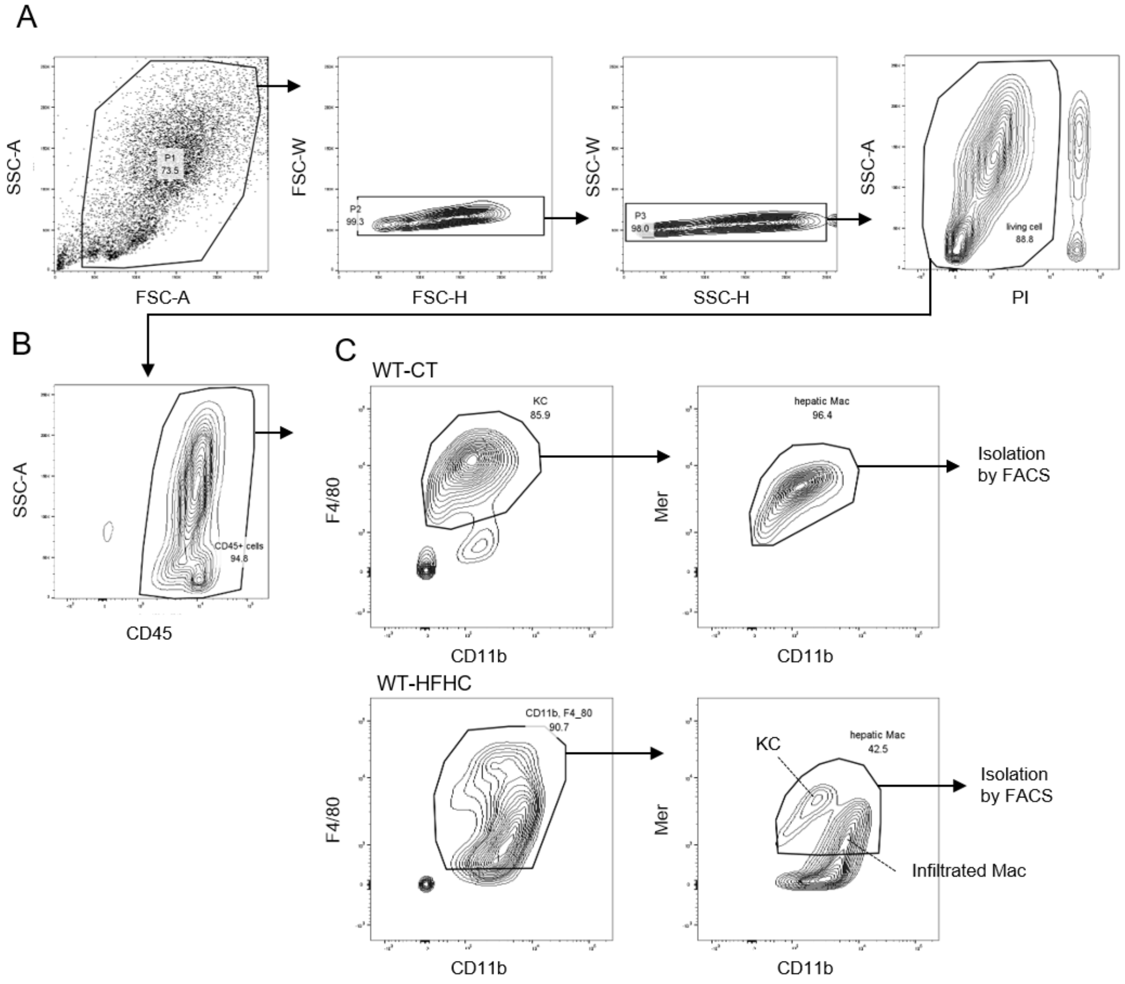

Figure S1 Fluorescence-activated cell sorting (FACS) for the isolation of hepatic macrophage. (A) Procedure for gating single and living cell populations in the liver tissues. (B) Gating of CD45+ cells (myeloid cells) in liver cells. (C) Representative images of CD11b+/F4/80+/Mer+ cells isolated from hepatic macrophages.

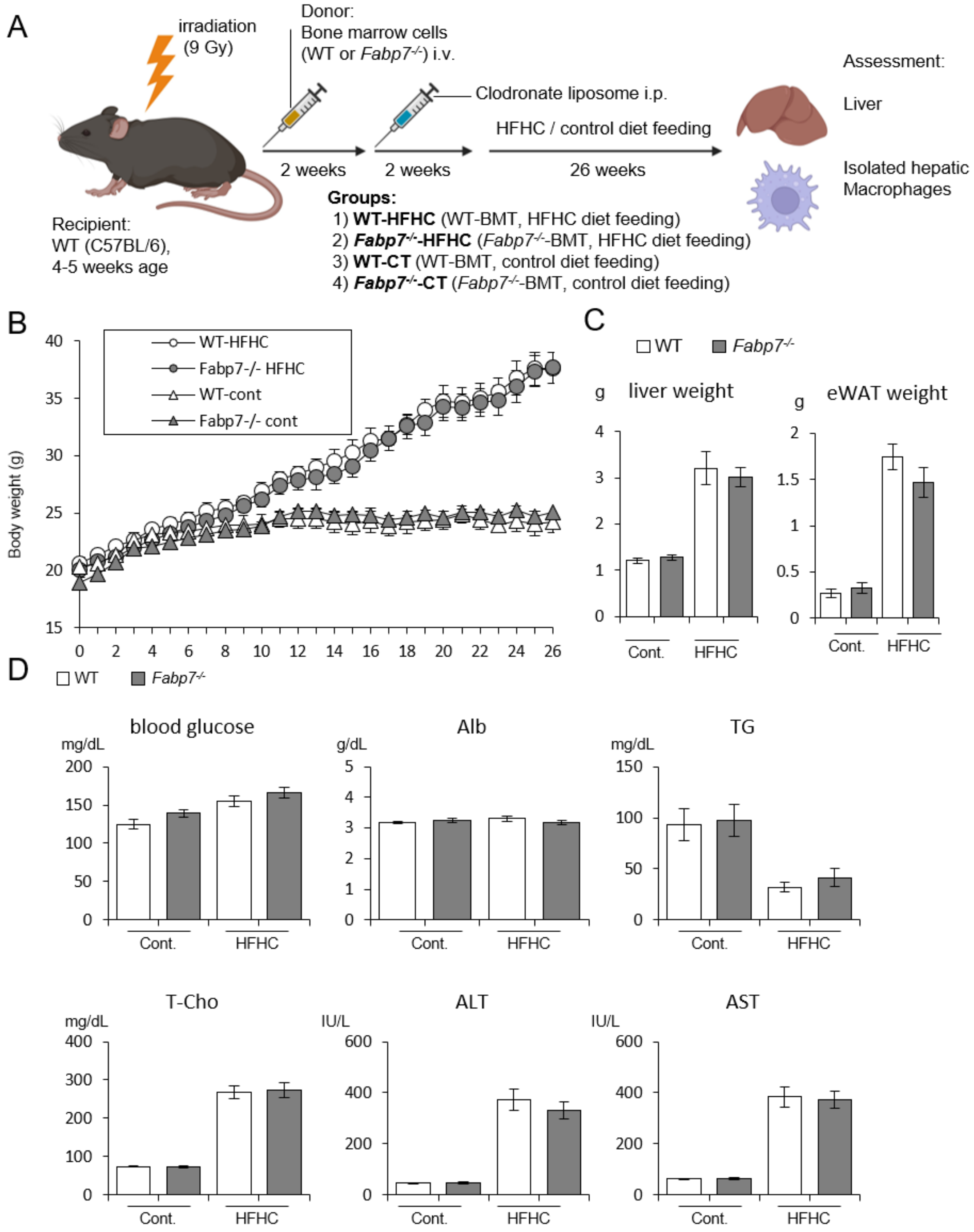

Figure S2 Metabolic dysfunction-associated steatohepatitis (MASH) induced by high-fat high-cholesterol (HFHC) diet feeding after bone marrow transplantation (BMT). (A) Procedure for establishing a MASH model post-BMT. For further details, please refer to

the Supplemental Materials and Methods. (B) Body weights of BMT mice in the control and HFHC diet groups (n=8/group). (C) Liver and epididymal white adipose tissue (eWAT) weights of BMT mice after intake of control or HFHC diet for 26 weeks (n=8/group). (D) Results of blood tests in BMT mice after intake of control or HFHC diet for 26 weeks (n=8/group).

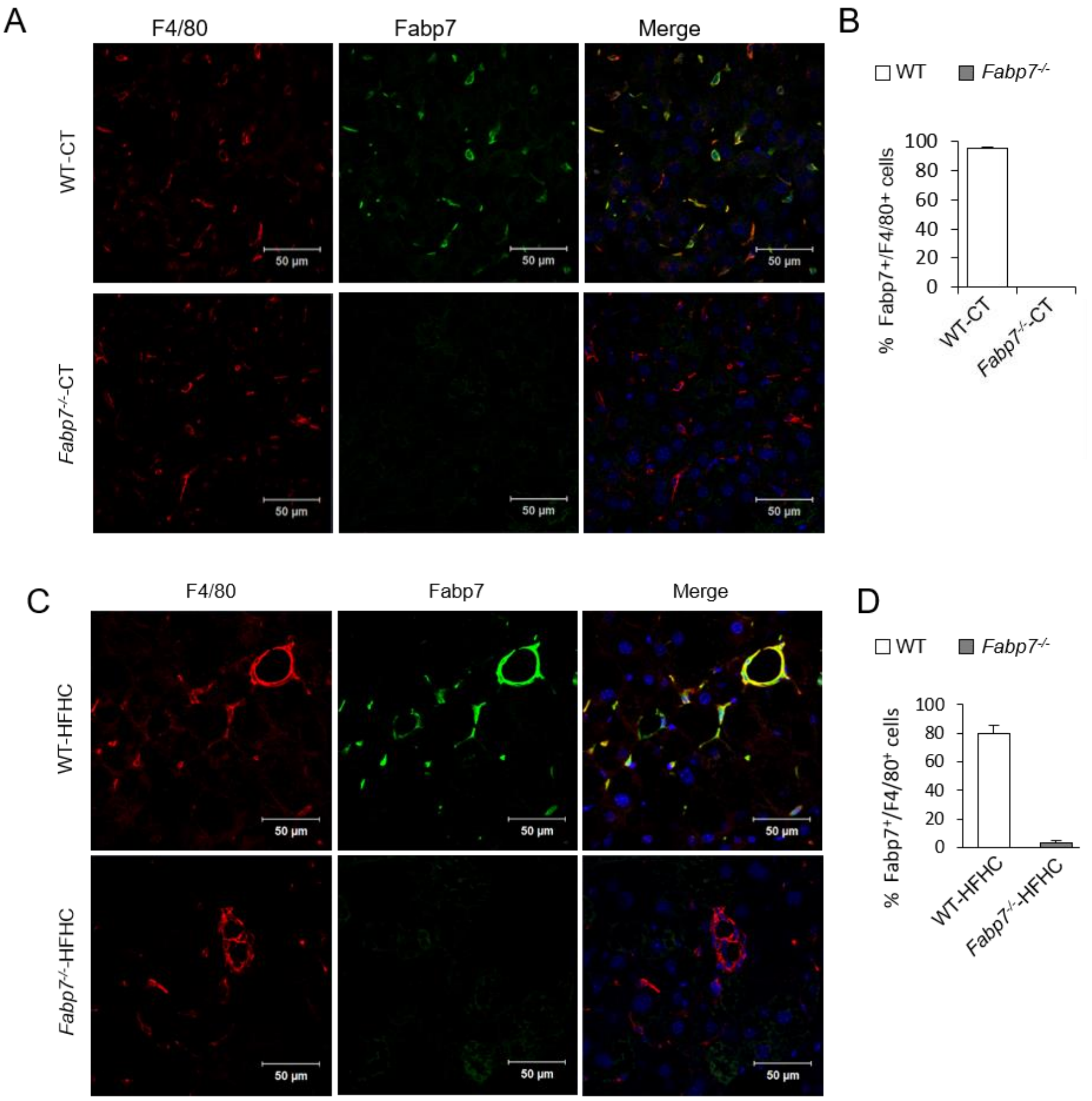

Figure S3. Hepatic macrophages do not express Fabp7 after *Fabp7*$^{-/-}$-BMT. (A) Representative images of the fluorescent immunostaining (F4/80 [red]/FABP7 [green]/ 4',6-diamidino-2-phenylindole [DAPI; blue]) of the liver tissues of wild-type (WT) and *Fabp7*$^{-/-}$-BMT mice after 26 weeks of control diet. (B) Percentage of Fabp7+ of F4/80+ cells in liver tissues (n=4/group). (C) Representative images of fluorescent immunostaining (F4/80 [red]/FABP7 [green]/DAPI [blue]) of the liver tissues of WT- and

Fabp7$^{-/-}$-BMT mice after 26 weeks of HFHC diet. (D) Percentage of Fabp7+ cells among F4/80+ cells in liver tissues (n=6/group).

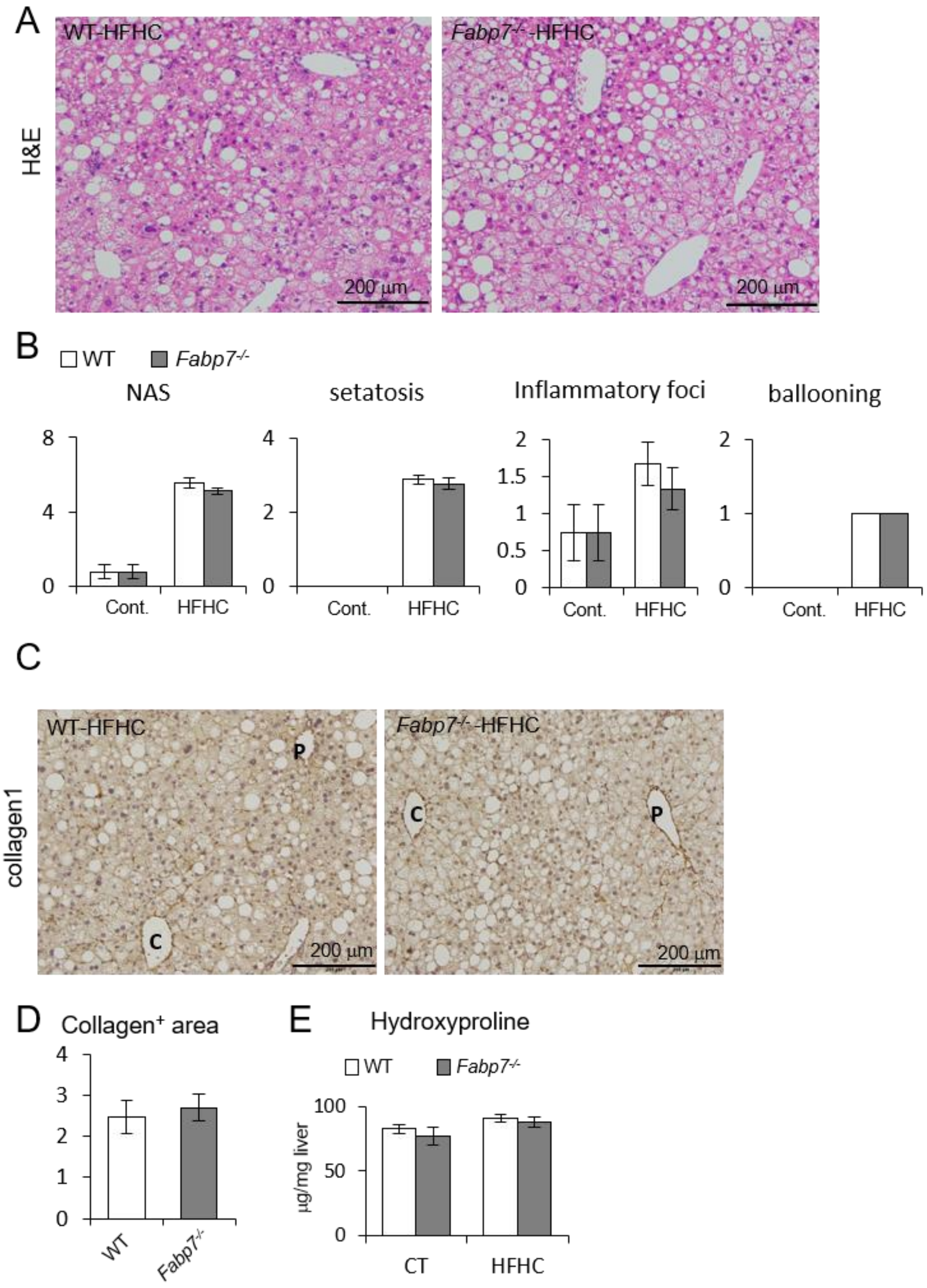

Figure S4 Fabp7 in hepatic macrophages does not affect liver damage induced by HFHC diet. (A) Representative images of the hematoxylin and eosin (H&E) staining of liver tissues of WT- or *Fabp7*$^{-/-}$-BMT mice after HFHC diet intake for 26 weeks. (B) NAFLD activity score (NAS) in the liver tissues of WT- or *Fabp7*$^{-/-}$-BMT mice after HFHC diet

intake for 26 weeks determined via histological assessment (n=8/group). (C) Representative images of collagen 1 immunostaining in the liver tissues of WT- or *Fabp7*$^{-/-}$-BMT mice after HFHC diet intake for 26 weeks. (D) Percentage of collagen1-positive areas in the liver tissues of WT- or *Fabp7*$^{-/-}$-BMT mice after HFHC diet intake for 26 weeks (n=8/group). (E) Hydroxyproline content in the liver tissues of WT- or *Fabp7*$^{-/-}$-BMT mice after control of HFHC diet intake for 26 weeks (n=4-8/group).

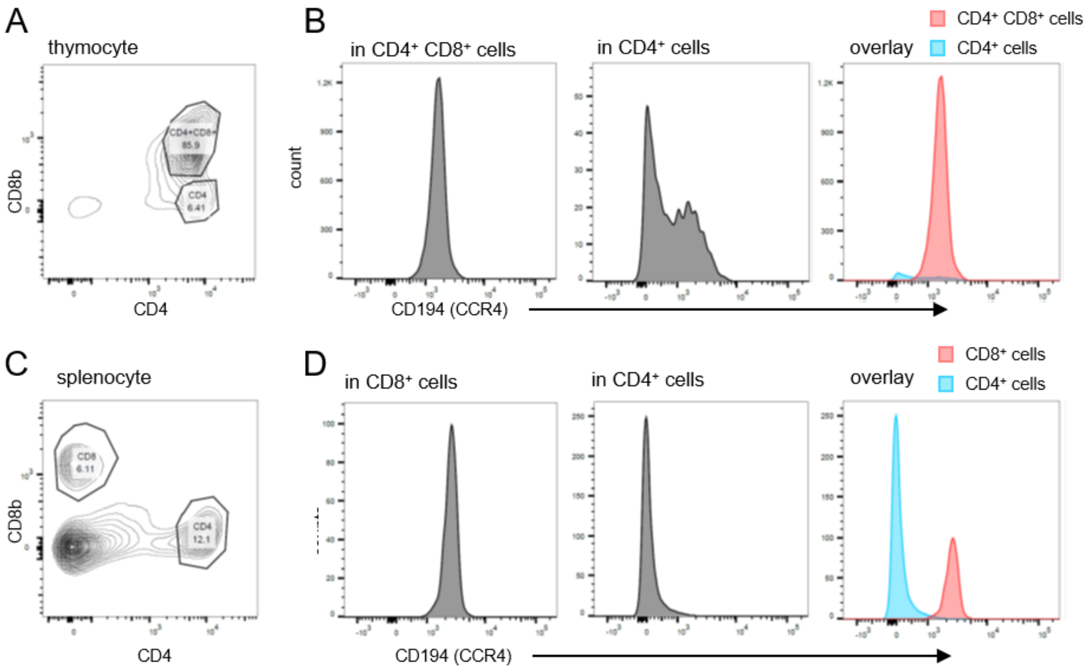

Figure S5 C-C motif chemokine receptor 4 **(**CCR4) expression levels in T cells isolated from thymus and spleen. (A) CD4 and CD8 expression levels in thymocytes after the elimination of dead or doublet cells. (B) CCR4 expression levels in $CD4^+$ cells and $CD4^+$ $CD8^+$ cells (C) CD4 and CD8 expression levels in splenocytes after elimination dead or doublet cells. (D) CCR4 expression levels in $CD4^+$ cells and $CD8^+$ cells.

References

[1] F. Kizuka, N. Tokuda, K. Takagi, Y. Adachi, L. Lee, I. Tamura, R. Maekawa, T. Taketani, H. Tamura, T. Suzuki, Y. Owada, N. Sugino, Involvement of bone marrow-derived vascular progenitor cells in neovascularization during formation of the corpus luteum in mice, Biol Reprod 87 (2012). https://doi.org/10.1095/biolreprod.112.099960.

[2] M. Furuhashi, R. Fucho, C.Z. Görgün, G. Tuncman, H. Cao, G.S. Hotamisligil, Adipocyte/macrophage fatty acid–binding proteins contribute to metabolic deterioration through actions in both macrophages and adipocytes in mice, Journal of Clinical Investigation (2008). https://doi.org/10.1172/jci34750.

[3] E.M. Brunt, D.E. Kleiner, L.A. Wilson, P. Belt, B.A. Neuschwander-Tetri, Nonalcoholic fatty liver disease (NAFLD) activity score and the histopathologic diagnosis in NAFLD: Distinct clinicopathologic meanings, Hepatology 53 (2011) 810–820. https://doi.org/10.1002/hep.24127.

[4] A. Di Stasi, B. De Angelis, C.M. Rooney, L. Zhang, A. Mahendravada, A.E. Foster, H.E. Heslop, M.K. Brenner, G. Dotti, B. Savoldo, T lymphocytes coexpressing CCR4 and a chimeric antigen receptor targeting CD30 have improved homing and antitumor activity in a Hodgkin tumor model, Blood 113 (2009) 6392–6402. https://doi.org/10.1182/blood-2009-03-209650.

[5] S. Kobayashi, T. Sakurai, T. So, Y. Shiota, A. Asao, H.T. Phung, R. Tanaka, T. Kawabe, T. Maruyama, E. Kanno, K. Kawakami, Y. Owada, N. Ishii, TNF Receptor–Associated Factor 5 Limits Function of Plasmacytoid Dendritic Cells by Controlling IFN Regulatory Factor 5 Expression, The Journal of Immunology 203 (2019) 1447–1456. https://doi.org/10.4049/jimmunol.1900188.
